# Transcriptome Analysis of an Anthracnose-Resistant Tea Plant Cultivar Reveals Genes Associated with Resistance to *Colletotrichum camelliae*

**DOI:** 10.1371/journal.pone.0148535

**Published:** 2016-02-05

**Authors:** Lu Wang, Yuchun Wang, Hongli Cao, Xinyuan Hao, Jianming Zeng, Yajun Yang, Xinchao Wang

**Affiliations:** 1 National Center for Tea Plant Improvement, Tea Research Institute, Chinese Academy of Agricultural Sciences, Hangzhou, China; 2 Key Laboratory of Tea Biology and Resources Utilization, Ministry of Agriculture, Hangzhou, China; 3 College of Horticulture, Northwest Agriculture and Forestry University, Yangling, 712100, Shaanxi, China; Hainan University, CHINA

## Abstract

Tea plant breeding is a topic of great economic importance. However, disease remains a major cause of yield and quality losses. In this study, an anthracnose-resistant cultivar, ZC108, was developed. An infection assay revealed different responses to *Colletotrichum* sp. infection between ZC108 and its parent cultivar LJ43. ZC108 had greater resistance than LJ43 to *Colletotrichum camelliae*. Additionally, ZC108 exhibited earlier sprouting in the spring, as well as different leaf shape and plant architecture. Microarray data revealed that the genes that are differentially expressed between LJ43 and ZC108 mapped to secondary metabolism-related pathways, including phenylpropanoid biosynthesis, phenylalanine metabolism, and flavonoid biosynthesis pathways. In addition, genes involved in plant hormone biosynthesis and signaling as well as plant-pathogen interaction pathways were also changed. Quantitative real-time PCR was used to examine the expression of 27 selected genes in infected and uninfected tea plant leaves. Genes encoding a MADS-box transcription factor, NBS-LRR disease-resistance protein, and phenylpropanoid metabolism pathway components (CAD, CCR, POD, beta-glucosidase, ALDH and PAL) were among those differentially expressed in ZC108.

## Introduction

Tea is a widely consumed non-alcoholic beverage and the tea plant (*Camellia sinensis* (L.) O. Kuntze) is a woody crop of worldwide economic importance [1]. The tea plant is susceptible to many bacterial, fungal and viral diseases. A wide variety of tea plant germplasms exist worldwide [2, 3], and breeding continues to produce new superior cultivars with more beneficial elements (e.g. free amino acid content, lower caffeine content and higher tolerance to abiotic or biotic stress).

The yield and quality of the tea plant can be severely affected by biotic stressors [4, 5]. Anthracnose disease (AD), which is caused by *Colletotrichum species* (sp.), causes severe plant damage and loss of crop yield and quality. AD is an economically devastating disease prevalent under warm and humid conditions [6, 7]. As the disease progresses, circular spots appear on the leaves, enlarge, become sunken, and then produce lesions with round edges [8]. AD can cause plant wilt, shoot death, and fragility. Unfortunately, many tea plant cultivars are highly susceptible to AD [7]. Developing resistant cultivars may be the most efficient and economical strategy to reduce the threat of the disease.

Understanding tea plant defense mechanisms is important for breeding resistant cultivars. However, little is known about the molecular mechanisms regulating the defense response in tea plants. Plants have evolved multiple defense signaling pathways to respond to environmental conditions and pathogen attack [9]. Secondary metabolites (classified as alkaloids, phenolics and terpenoids) are important in plant protection to attenuate the effects of abiotic and biotic stress. Phenylpropanoid compounds are precursors to a wide range of phenolic compounds (e.g. flavonoids, isoflavonoids, anthocyanins, plant hormones, phytoalexins, and lignins) that play a vital role in host defense [10–12]. Phenylpropanoid pathway-associated genes comprise a large family involved in plant responses to various stressors. For example, induction of the phenylpropanoid pathway increases mango resistance to *Ceratocystis fimbriata* infection [13]. Furthermore, induction of four enzymes in the phenylpropanoid pathway is important for resistance to ascochyta blight disease in the chickpea [14]. Phenylalanine ammonia-lyase (PAL) is the first enzyme of phenylpropanoid metabolism. PAL controls a key branch point in the pathway of flavonoid biosynthesis, which generates antimicrobial phytoalexins and plays important roles in the response to biotic and abiotic stress [15]. Therefore, early or high PAL activity in response to infection is considered an indicator of pathogen resistance. For example, in the pepper plant, PAL1 enzymatic activity in the phenylpropanoid pathway acts as a positive regulator of salicylic acid (SA)-dependent defense signaling to combat microbial pathogens [16]. Furthermore, functional analysis of tobacco PAL showed that PAL-suppressed transgenic tobacco has undeveloped systemic acquired resistance and reduced SA levels in leaves [17].

Lignin is a major end-product of phenylpropanoid metabolism. Down-regulating lignin pathway genes alters metabolic flux and affects the biosynthesis of other secondary compounds [18]. Cinnamoyl-CoA reductase (CCR) and cinnamyl alcohol dehydrogenase (CAD) catalyze the first and last steps in the lignin biosynthetic pathway, respectively [19]. Down-regulating CCR affects the composition of soluble phenolics by increasing various ferulate derivatives in *Arabidopsis*, poplar, and tobacco [20–23]. A similar pattern was observed in CAD-downregulated tobacco [24]. Reducing lignin content may trigger signaling pathways that activate stress genes [25]. Thus, lignin downregulation can alter phenylpropanoid metabolism, thereby affecting the biosynthesis of secondary compounds that play important roles in plant-environment interactions [18].

Many studies have investigated the relationship between AD and plants. In an AD-resistant muscadine cultivar, the chalcone synthase (*CHS*) gene in the phenylpropanoid pathway, which condenses three malony-CoA molecules with cinnamoyl-CoA to produce chalcone, may contribute to resistance [26]. Louime and colleagues observed differences in gene transcription between AD-susceptible and -tolerant grapevine cultivars, and identified many genes (CHS, stilbene synthase, polygalacturonase-inhibiting protein, chitinase and lipid transfer protein) that are only expressed in tolerant cultivars in response to anthracnose pathogen infection [26]. Vasanthaiah *et al*. demonstrated that chitinase and stilbene synthase genes were more rapidly expressed in tolerant cultivars after inoculation with an anthracnose pathogen [27]. In addition to PAL and CHS, other enzymes (e.g. chalcone isomerase (CHI) and dihydroflavonol 4-reductase (DFR)), which make up the flavonoid biosynthesis pathway, were also differentially expressed after inoculation in resistant and susceptible grapevines [8].

In this study, we characterized the anthracnose-resistant tea plant cultivar Zhongcha 108 (ZC108). ZC108 was produced by irradiating the offspring of Longjing 43 (LJ43) [28]. These two cultivars demonstrated different levels of resistance to *Colletotrichum camelliae* infection. Additionally, the two cultivars had many different characteristics, including bud sprouting time, leaf shape, and plant architecture. A 4 × 44 K custom microarray analysis revealed 2,453 differentially expressed genes between ZC108 and LJ43. These include genes involved in transcriptional regulation, phenylpropanoid metabolism, flavonoid biosynthesis, plant hormone signal transduction, and plant-pathogen interaction. Furthermore, several genes that were differentially regulated post-infection between ZC108 and LJ43 were identified by qRT-PCR.

## Material and Methods

### Plant material and sample preparation

Two tea plant (*Camellia sinensis* (L.) O. Kuntze) cultivars, LJ43 and ZC108, were used in this study. Plants were grown in the field at the Tea Research Institute of the Chinese Academy of Agricultural Sciences (TRI, CAAS, N 30°10', E 120°5'), Hangzhou, China. ZC108 was selected from the offspring of LJ43 cuttings under Co^60^γ-ray radiation [28]. The cutting shoots of LJ43 were irradiated by Co^60^γ-ray using 9.5 Gy irradiation dose in 1986, then the mutant offsprings (M1) were cuttage propagated for single plant selection. After 24 years breeding procedure, one new line was selected out and it performed early sprouting time, anti-anthracnose disease and high quality for processing green tea compared to LJ43. Then the line was named as ZC108 and registered as a new cultivar in China in 2010. And the parentage between LJ43 and ZC108 was identified by SSR markers and microarray method [29, 30].

For leaf measurement, mature leaves of 5-year-old LJ43 and ZC108 were randomly collected from more than 20 tea plants. The width and length of the leaves was measured. Sprouting time of the first new shoot (one leaf and one bud) was observed in spring of 2013 and 2014. For microarray analysis, the fresh shoots (two leaves and one bud) were sampled from the 5-year-old plants grown in the field [30]. Because the AD always occur at mature leaves, so for infection assay, the third healthy mature leaf from the top of each cultivar was collected from 5-year-old tea plants for *in vitro* inoculation. For qRT-PCR analysis, 3-year-old plants were grown in the green house for 1 month before *in vivo* inoculation, after inoculation, the mature leaves of LJ43 and ZC108 at 0, 24 and 72 h post infection were sampled. Three independent biological replicates were performed.

### Pathogen inoculations

Two strains of *Colletotrichum camelliae* were used in this study. They were isolated from diseased tea plants in Shaanxi (N 107°67', E 32°96') and Zhejiang (N 30°10', E 120°5'), China, and are indicated as *Colletotrichum camelliae*-1 and -2, respectively. The two strains were isolated from private land and did not require permits. *Colletotrichum camelliae*-1 and -2 were cultured on potato dextrose agar at 25°C under 12h light/12h dark for 7 days. Conidia were harvested and suspended in sterile water, and conidia concentrations were adjusted to 1**×**10^5^ ml^-1^.

For pathogenicity tests, LJ43 and ZC108 were inoculated with *Colletotrichum camelliae*. Leaves were surface-sterilized with 75% ethanol and sterile distilled water. The leaves were wounded with a sterile needle, and 20μl conidial suspension were applied to the wound. Inoculated leaves were placed in wet cotton wool to maintain sample moisture and incubated at 30°C. Inoculation experiments were conducted independently and performed in triplicate.

### Microarray data analysis

For the microarray assay, total RNA was purified using the RNeasy Mini Kit (QIAGEN, Hilden, Germany). The custom microarray used in this study was developed by our laboratory as previously described [30]. All microarray data were deposited into the Gene Expression Omnibus (GEO) database under Accession Number GSE52255 (Release date 2015-1-1).

Raw data were obtained and analyzed as previously described [31]. Differential gene expression was defined as >2-fold change (FC) with *P*< 0.05. To identify biological pathways, genes were annotated with the corresponding enzyme commission (EC) numbers from BLASTX alignments against the Kyoto Encyclopedia of Genes and Genomes (KEGG) database [32, 33]. Pathways were selected using Fisher’s exact test (*P*< 0.05, false discovery rate (FDR) < 0.05) [34]. Gene ontology (GO) terms were assigned based on the best BLASTx hit from the NR database using Blast2GO software [35].

### Quantitative Real-Time PCR (qRT-PCR)

Total RNA was extracted from ZC108 and LJ43 leaf samples (0.5-1g) at 0, 24 and 72 hours post inoculation with *Colletotrichum camelliae*, as previously described [36]. RNA samples (5 μg) were treated with RNase-free DNase I (Invitrogen, Carlsbad, CA, USA) to remove residual genomic DNA, and then used for cDNA synthesis with SuperScript^®^ III Reverse Transcriptase (Invitrogen). Reaction mixtures were diluted 1:40 with distilled water and used as templates for qRT-PCR. Primer information is listed in S1 Table. Quantitative assays were performed in triplicate on each cDNA sample, and transcript levels were calculated relative to the level of polypyrimidine tract-binding protein (PTB) using the 2^-ΔΔCt^ formula [37, 38]. All data are presented as mean ± SD (*n* = 3).

Hierarchical clustering analysis of the expression data was carried out using Cluster 3.0. QRT-PCR data of LJ43 and ZC108 were normalized to LJ43 0h time point. Microarray data of ZC108 were normalized to microarray data of LJ43. The color scale represents log2 expression values and the expression levels presented in heatmap were log2-based.

### Statistical analysis of the data

Data are expressed as the mean±SD from three independent biological replicates. Significance was determined via one-way analysis of variance followed by the post-hoc least significant difference (LSD) *t*-test (*P* < 0.05).

## Results

### Phenotypic performance of ZC108

**Fig 1:**
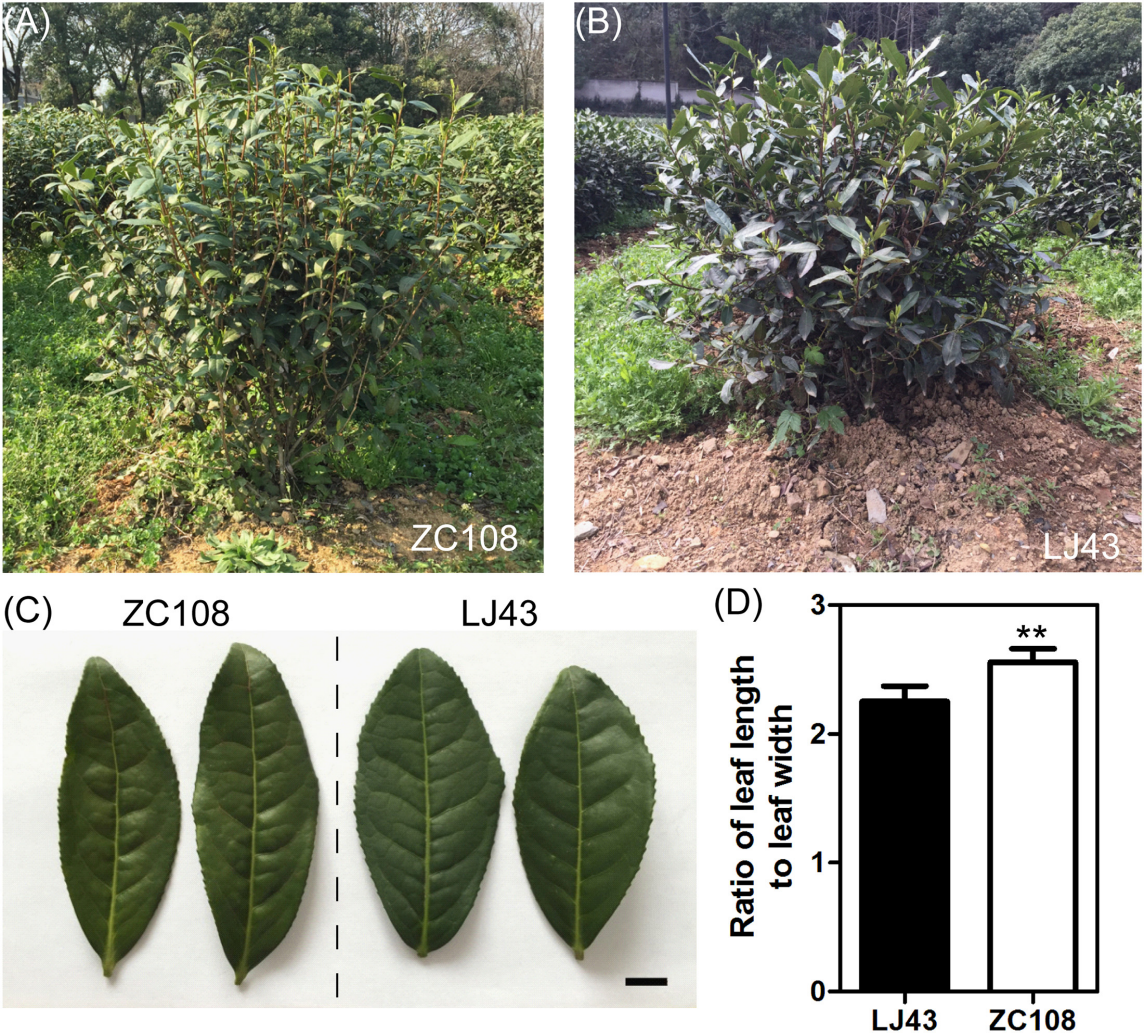
Growth performance and leaf morphology of LJ43 and ZC108 grown in the field. (A, B) Growth performance of ZC108 (A) and LJ43 (B) in the field in spring. (C) Mature leaf morphology of LJ43 and ZC108, bar = 1 cm. (D) Length:width ratio in mature leaves of LJ43 and ZC108. Data shown as the mean ±SD (*n* = 35). **P < 0.01 vs LJ43.

ZC108, the radiation-induced mutant of LJ43, showed many phenotypic differences compared to LJ43. One difference is bud sprouting time. Early sprouting in the spring is an attractive characteristic to tea growers because it confers higher economic value. Therefore, one goal of tea plant breeding is developing cultivars with earlier sprouting time. ZC108 sprouted earlier than LJ43 in the spring (Table 1). ZC108 and LJ43 also had different plant architecture (Fig 1A and 1B). Under normal conditions, the branch angle of ZC108 was smaller than that of LJ43 (Fig 1A and 1B). Shoot branching controls the yield of tea plant; therefore, ZC108 had higher yield than LJ43 [28]. Additionally, ZC108 leaves had a higher length to width ratio than LJ43 leaves (Fig 1C and 1D).

**Table 1:** Sprouting date of ZC108 and LJ43 cultivated in two areas in spring of 2013 and 2014.

	Date of one leaf and one bud (Area 1)		Date of one leaf and one bud (Area 2)	
Cultivars	2013	2014	2013	2014
ZC108	23/3	29/3	2/4	1/4
LJ43	2/4	31/3	6/4	3/4

### ZC108 is an anthracnose-resistant tea plant cultivar

**Fig 2:**
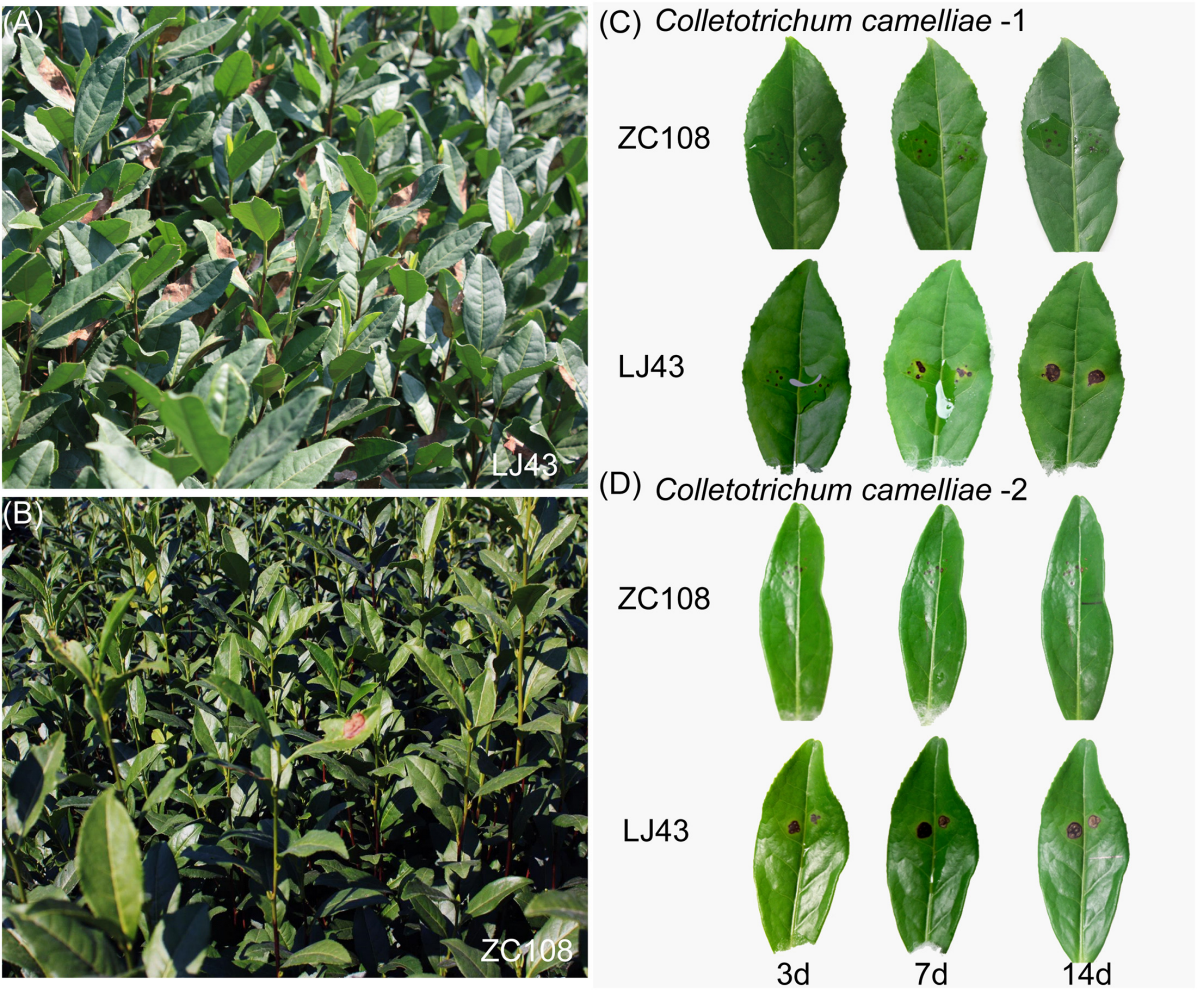
Altered disease resistance of tea leaves to AD in ZC108. (A, B) Leaf symptoms of LJ43 (A) and ZC108 (B) after suffering from AD in the field. (C, D) Differential disease resistance of tea leaves to *Colletotrichum camelliae* (1×10^5^ ml^-1^) infection in LJ43 and ZC108. Two strains of *Colletotrichum camelliae* pathogens were used. *Colletotrichum camelliae*-1 and -2 were isolated from diseased tea plant leaves in Shaanxi and Zhejiang, respectively. Photos were taken on 3, 7 and 14 days post inoculation.

In the field, ZC108 exhibited higher resistance than LJ43 to AD. LJ43 plants showed disease symptoms on their leaves, but ZC108 plants appeared healthy (Fig 2A and 2B). To confirm the differential response to AD, leaves were inoculated *in vitro*. Both LJ43 and ZC108 were inoculated with two strains of *Colletotrichum camelliae*. Disease symptoms were observed on leaves 3, 7, and 14 days after inoculation. Anthracnose resistance was examined in the third mature leaf from the top of each cultivar. After inoculation, ZC108 symptoms appeared as tiny spots, and did not worsen over the time. However, LJ43 developed larger, sunken spots that produced lesions with round edges (Fig 2C and 2D). These results indicate that, compared to LJ43, ZC108 is more resistant to *Colletotrichum camelliae* infection.

### Microarray analysis

**Fig 3:**
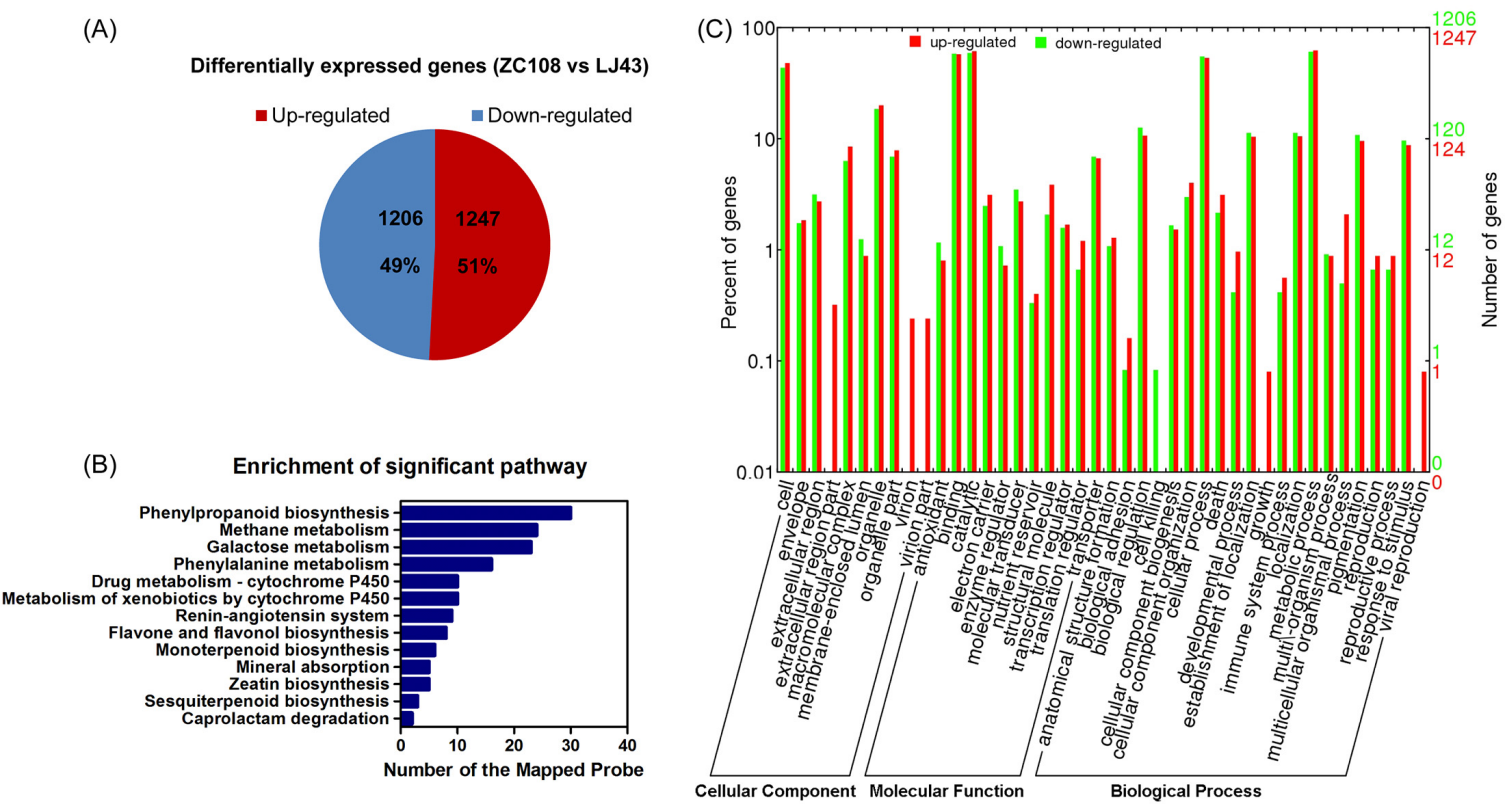
Differential expression analyses in ZC108 and LJ43. **(A)** Number of significantly differentially expressed genes (*P* < 0.05, fold change (FC) > 2) in ZC108 compared with LJ43. **(B)** Significantly (*P* < 0.05, FDR < 0.05) enriched pathways (based on KEGG) among the 2,453 differentially expressed genes. **(C)** GO classification of the 2,453 differentially expressed genes.

To investigate the mechanism underlying the phenotype of ZC108 plants, differences in gene expression between ZC108 and LJ43 were investigated using a *Camellia sinensis* custom microarray [30]. 2,453 genes were differentially expressed, 1,247 (51%) of which were up-regulated and 1,206 (49%) down-regulated in ZC108 (Fig 3A). Further analysis showed that genes mapped to 236 pathways (S2 Table). Notably, 13 pathways showed significant enrichment (*P* < 0.05, FDR < 0.05) (Fig 3B).

The most significantly enriched pathway was phenylpropanoid biosynthesis, suggesting that genes related to phenylpropanoid metabolism may play important roles in the phenotype of ZC108 (Fig 3B). Genes involved in lignin biosynthesis (including PAL (EC:4.3.1.24), CAD (EC:1.1.1.195) and CCR (EC:1.2.1.44)), as well as peroxidase (EC:1.11.1.7, POD), beta-glucosidase (EC:3.2.1.21) and aldehyde dehydrogenase (EC:1.2.1.68, ALDH) genes, showed significant changes in ZC108. All ALDH and PAL probes were up-regulated, while most POD, all CCR, and all beta-glucosidase probes were down-regulated in ZC108 (Table 2). Notably, CAD, CCR, POD, beta-glucosidase, ALDH and PAL are important in the defense response and are related to plant pathogen resistance [39–45].

**Table 2:** Differentially expressed genes involved in phenylpropanoid biosynthesis (FC > 2, *P* < 0.05).

Probe name	ZC108 vs LJ43	Genes
**Up-regulated**		
CUST_10940_PI428262022	137.69	CAD
CUST_6622_PI428262022	90.23	ALDH
CUST_33801_PI428262022	13.05	CAD
CUST_506_PI428262022	6.89	ALDH
CUST_3374_PI428262022	5.67	PAL
CUST_21655_PI428262014	4.52	PAL
CUST_35286_PI428262014	4.45	ALDH
CUST_20888_PI428262022	3.47	ALDH
CUST_25079_PI428262022	2.86	POD
CUST_1515_PI428262022	2.75	POD
CUST_11962_PI428262022	2.57	caffeoyl-CoA O-methyltransferase (CCoAOMT)
CUST_20317_PI428262022	2.29	PAL
CUST_1517_PI428262022	2.06	POD
**Down-regulated**		
CUST_42643_PI428262014	-6.05	POD
CUST_36442_PI428262014	-5.36	CAD
CUST_7937_PI428262014	-5.24	POD
CUST_2483_PI428262014	-4.03	CCR
CUST_33994_PI428262014	-3.63	POD
CUST_13516_PI428262014	-3.06	beta-glucosidase
CUST_41896_PI428262022	-2.85	POD
CUST_54961_PI428262014	-2.77	beta-glucosidase
CUST_6244_PI428262022	-2.74	POD
CUST_6222_PI428262014	-2.73	POD
CUST_10739_PI428262022	-2.64	CAD
CUST_371_PI428262014	-2.53	beta-glucosidase
CUST_50442_PI428262014	-2.47	POD
CUST_9847_PI428262014	-2.27	POD
CUST_16981_PI428262014	-2.10	4-coumarate—CoA ligase
CUST_40717_PI428262022	-2.09	CCR
CUST_389_PI428262014	-2.05	beta-glucosidase

In addition, genes related to plant hormone signaling (Table 3) and plant-pathogen interaction (Table 4) were affected. Among the differentially expressed hormone-related genes, the majority were auxin-related (Table 3). Differences in sprouting time and plant architecture between LJ43 and ZC108 may be due to these pathways. In addition, plant-pathogen interaction related genes were differentially expressed, including nucleotide-binding site (NBS) and leucine-rich repeat (LRR) resistance proteins and heat shock protein (HSP) [46–48], which may account for the different AD resistance of the two cultivars (Table 4).

**Table 3:** Differentially expressed genes related to plant hormone biosynthesis and signaling pathways (FC > 2, *P* < 0.05).

Probe name	ZC108 vs LJ43	Definition
**Auxin related**		
CUST_50500_PI428262014	-6.16	auxin influx carrier (AUX1 LAX family)
CUST_11577_PI428262014	-4.74	SAUR family protein
CUST_15249_PI428262022	-3.08	auxin responsive GH3 gene family
CUST_15250_PI428262014	-2.53	auxin responsive GH3 gene family
CUST_14183_PI428262014	-2.46	SAUR family protein
CUST_2448_PI428262014	-2.30	auxin-responsive protein IAA
CUST_20521_PI428262014	-2.28	auxin response factor
CUST_18314_PI428262022	-2.12	SAUR family protein
CUST_1953_PI428262022	-2.11	auxin-responsive protein IAA
CUST_16624_PI428262022	2.96	SAUR family protein
CUST_12_PI428262022	2.09	auxin response factor
CUST_10678_PI428262014	2.05	auxin response factor
CUST_26069_PI428262014	2.05	auxin-responsive protein IAA
**ABA related**		
CUST_12149_PI428262014	-25.2	ABA receptor PYR/PYL family
CUST_20599_PI428262022	-3.44	brassinosteroid insensitive 1-associated receptor kinase 1
CUST_7855_PI428262022	-2.73	ABA responsive element binding factor
CUST_10100_PI428262014	-2.17	ABA responsive element binding factor
**Jasmonate related**		
CUST_8238_PI428262014	-2.44	jasmonate ZIM domain-containing protein
CUST_13372_PI428262014	4.26	jasmonate ZIM domain-containing protein
CUST_3660_PI428262022	3.21	jasmonate ZIM domain-containing protein
CUST_7185_PI428262022	2.93	jasmonate ZIM domain-containing protein
**Zeatin related**		
CUST_13969_PI428262022	13.38	cis-zeatin O-glucosyltransferase
CUST_6601_PI428262014	-12.43	tRNA dimethylallyltransferase
CUST_17809_PI428262014	-9.93	cis-zeatin O-glucosyltransferase
CUST_19864_PI428262014	-2.90	cytokinin dehydrogenase
CUST_37226_PI428262014	-2.68	cytokinin dehydrogenase
**Other hormones**		
CUST_548_PI428262014	-2.44	ethylene receptor
CUST_2735_PI428262014	-2.13	protein brassinosteroid insensitive 1
CUST_809_PI428262022	-2.11	DELLA protein
**Regulation**		
CUST_42121_PI428262022	7.32	histidine-containing phosphotransfer peotein
CUST_3858_PI428262022	3.63	serine/threonine-protein kinase SRK2
CUST_48154_PI428262022	2.35	transcription factor TGA
CUST_54248_PI428262022	2.35	transcription factor MYC2

**Table 4:** Differentially expressed genes mapped to the plant-pathogen interaction pathway (KO04626) in KEGG (FC > 2, *P* < 0.05).

Probe name	ZC108 vs LJ43	Definition
**Up-regulated**		
CUST_18562_PI428262022	14.79	CC-NBS-LRR resistance protein
CUST_54537_PI428262014	9.15	NBS-LRR resistance protein
CUST_50104_PI428262022	7.39	CC-NBS-LRR resistance protein
CUST_13372_PI428262014	4.26	jasmonate ZIM domain-containing protein
CUST_34812_PI428262022	4.01	NBS-LRR resistance protein (RPS2)
CUST_53112_PI428262014	3.92	calcium-dependent protein kinase
CUST_14941_PI428262022	3.57	NBS-LRR resistance protein
CUST_47812_PI428262022	3.52	cyclic nucleotide gated channel
CUST_15181_PI428262022	3.34	NBS-LRR resistance protein (RPS2)
CUST_19468_PI428262022	3.24	NBS-LRR disease resistance protein (RPM1)
CUST_3660_PI428262022	3.21	jasmonate ZIM domain-containing protein
CUST_20165_PI428262022	2.98	CC-NBS-LRR resistance protein
CUST_7185_PI428262022	2.93	jasmonate ZIM domain-containing protein
CUST_19115_PI428262022	2.91	transcription factor MYC2
CUST_17805_PI428262022	2.4	disease resistance protein RPM1 (NBS-LRR)
CUST_54248_PI428262022	2.35	transcription factor MYC2
CUST_56099_PI428262014	2.26	glycerol kinase
CUST_20394_PI428262022	2.24	NBS-LRR resistance protein
CUST_19763_PI428262014	2.18	chitinase
CUST_14991_PI428262022	2.14	molecular chaperone HtpG
CUST_20060_PI428262022	2.14	respiratory burst oxidase
CUST_23265_PI428262014	2.11	suppressor of G2 allele of SKP1
CUST_7334_PI428262022	2.09	cyclic nucleotide gated channel
CUST_7592_PI428262014	2.04	suppressor of G2 allele of SKP1
**Down-regulated**		
CUST_18102_PI428262014	-10.68	heat shock protein 90kDa beta
CUST_36014_PI428262014	-6.74	NBS-LRR disease resistance protein (RPM1)
CUST_26291_PI428262014	-4.09	heat shock protein
CUST_9744_PI428262022	-3.55	mitogen-activated protein kinase kinase kinase 1
CUST_52781_PI428262014	-3.52	cyclic nucleotide gated channel
CUST_40627_PI428262014	-3.5	cyclic nucleotide gated channel
CUST_20599_PI428262022	-3.44	brassinosteroid insensitive 1-associated receptor kinase 1
CUST_51979_PI428262022	-2.61	cyclic nucleotide gated channel
CUST_8238_PI428262014	-2.44	jasmonate ZIM domain-containing protein
CUST_28392_PI428262014	-2.30	CC-NBS-LRR resistance protein
CUST_7836_PI428262014	-2.27	Calmodulin
CUST_19961_PI428262022	-2.16	Calmodulin
CUST_35507_PI428262022	-2.08	NBS-LRR resistance protein
CUST_49241_PI428262014	-2.06	CC-NBS-LRR resistance protein

We performed a GO functional annotation of differentially-expressed genes. Genes were categorized as belonging to biological process (BP), molecular function (MF), and cellular component (CC) functional groups. Within each functional group, the largest categories were metabolic and cellular processes (BP), binding and catalytic activity (MF), and cell and organelle (CC) (Fig 3C). Microarray analysis also revealed that 7 probe sets corresponded to transcription factors (TFs) encoding AP2/ERF, MYB, ZF-HD, C2H2, MDAS, REM and bZIP family proteins (Table 5). *Arabidopsis* homologs of these TFs are involved in plant development and stress response.

**Table 5:** Classification of differentially expressed genes encoding transcription factors.

Probe	ZC108 vs LJ43	Arabidopsis TF	Homologs	Target processes	Similarity	Matching Length (bp)	E value
CUST_3691_PI428262014	-2.25	AT1G15360	AP2/ERF	Pathogenesis-related, wax biosynthesis, drought tolerance	82.98	141	4.00E-30
CUST_767_PI428262022	2.68	AT3G01140	MYB	Plant development, trichome branching	80.44	501	5.00E-105
CUST_4560_PI428262022	3.12	AT2G02540	ZF-HD	Expressed in vascular tissue	82.72	162	3.00E-35
CUST_8204_PI428262022	3.1	AT5G16470	C2H2 (MBS2)	Stress response, ROS signaling	76.39	216	5.00E-27
CUST_33289_PI428262022	2.42	AT4G11880	MADS (AGL14)	Agamous-like	80.93	236	4.00E-41
CUST_1834_PI428262022	2.42	AT3G53310	REM	Plant development	100	28	3.00E-07
CUST_4471_PI428262014	-2.98	AT5G06950	bZIP (TGA2)	Plant pathogen interaction, regulate PR gene	85	99	9.00E-16

Note: Genes from the tea plant showing similarity to *Arabidopsis* homologs higher than 75% with e value < 1e-5 are listed. The target process was described according to the TAIR database.

### Validation of differentially expressed genes

**Fig 4:**
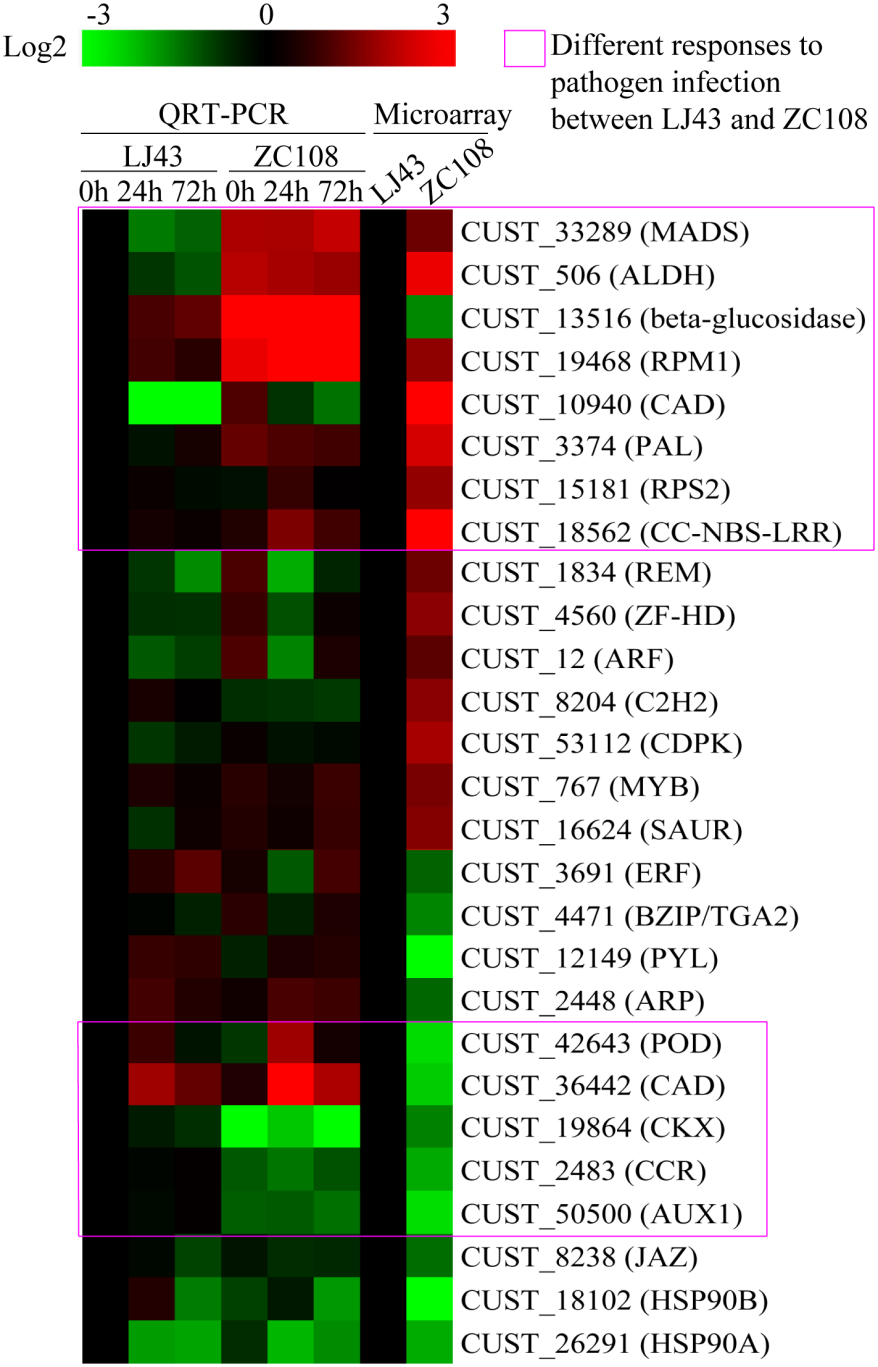
Validation and expression analysis of 27 genes in response to *Colletotrichum camelliae* infection in leaves of LJ43 and ZC108. Gene expression analysis were examined by qRT-PCR using cDNA from leaves of three-year-old LJ43 and ZC108 after inoculated by *Colletotrichum camelliae* for 0, 24 and 72 h. All of the qRT-PCR values were expressed relative to the expression level of LJ43-0h (control-set to 1.0). The data of ZC108 from microarray was expressed relative to the transcript abundance of LJ43 from microarray (control-set to 1.0). The color scale represents log2 expression values and the expression levels presented in heatmap were log2-based. All data are shown as the mean ± SD (*n* = 3).

To verify the microarray results, 27 genes from Tables 2–5 that were differentially expressed in ZC108 were analyzed using qRT-PCR. The microarray and qRT-PCR results are compared in Fig 4. Most of the selected genes analyzed by qRT-PCR at 0h showed an expression pattern consistent with the microarray data. About 6 genes including beta-glucosidase, C2H2, ERF, bZIP, ARP, CAD (CUST_36442) showed inconsistent regulation between qRT-PCR and microarray. This discrepancy may be attributed to several factors, including the different sensitivities of the analytical methods and the different samples used as templates in qRT-PCR and microarray.

To investigate molecular responses to pathogen infection in resistant ZC108 and wildtype LJ43 cultivars, leaves from both cultivars were infected with *Colletotrichum camelliae* and the expression of selected genes was measured. Expression of many of the selected genes changed in response to pathogen infection (Fig 4). Moreover, compared to LJ43, some genes also significantly changed in abundance at 24h and 72h post-infection in ZC108. These genes include a MADS-box transcription factor, 3 NBS-LRR family genes, plant hormone signal transduction-related genes (cytokinin dehydrogenase (CKX) and auxin-responsive protein IAA (AUX1)), and phenylpropanoid biosynthesis-related genes (CAD, CCR, POD, beta-glucosidase, ALDH and PAL) (Fig 4 and S1–S4 Figs). Notably, the expression of genes encoding defense enzymes (e.g. CAD, POD, PAL, ALDH and beta-glucosidase), the disease resistance protein NBS-LRR, and MADS-box transcription factor were significantly up-regulated in ZC108 after infection. Genes whose expression changed after infection in ZC108 may underlie tea plant responses against *Colletotrichum camelliae* infection.

Additionally, we found that the hormone signal transduction related genes ARF, SAUR, CKX, AUX1 and the transcription factor C2H2 showed significant differences in transcript abundance in ZC108 compared to LJ43 independent of pathogen infection condition (Fig 4, S1 and S3 Figs). These genes may therefore be responsible for the differential development and morphology between the two cultivars.

## Discussion

Compared to its parent cultivar LJ43, the irradiated offspring cultivar ZC108 showed important phenotypic differences. These include the important agronomic characteristics plant architecture, bud sprouting time, and AD resistance. These changes were beneficial to the economic value of the cultivar [28]. In this study, we used microarrays to investigate which genes contribute to the phenotypic differences between LJ43 and ZC108. Our results provide insight into the molecular mechanisms underlying these changes, especially the response to *Colletotrichum camelliae* infection.

Early sprouting time in spring confers higher economic tea plant value in China. Therefore, producing cultivars with early sprouting time is an important goal of tea plant breeders. Plant hormone signaling is the most important of many factors influencing sprouting time. Many studies have shown that auxin (IAA), gibberellin (GA), abscisic acid (ABA) and cytokinins (e.g. zeatin) participate in sprouting progress [49, 50] and plant architecture [51–54]. In the tea plant, free GA and IAA increase before release from dormancy, suggesting they may promote sprouting [55, 56]. Moreover, we found that before tea plant bud sprouting, auxin and ABA content reach a peak and nadir, respectively (unpublished data). The microarray analysis results in this study show that most genes related to plant hormone biosynthesis and signaling pathways are down-regulated in ZC108 compared to LJ43 (Table 3). These results suggest that changes in biosynthesis and plant hormone signaling may underlie the differential sprouting time and plant architecture in ZC108.

Tea plant AD severely affects plant health and tea production [7]. Most green tea plant cultivars are susceptible to AD, and therefore breeding anthracnose resistant cultivars is of high importance. According to performance and pathogenicity tests, ZC108 is an anthracnose-resistant cultivar. To acquire further insight into the molecular mechanisms underlying anthracnose resistance in ZC108, differentially expressed genes related to plant-pathogen interaction were identified by microarray. Plant hormones not only regulate growth and development, but also interact with the immune system [9, 57]. Reports indicate that all plant hormones can induce plant defense responses and participate in systemic immunity [58]. Many of the differentially expressed genes identified between ZC108 and LJ43 are implicated in the biosynthesis of plant hormones, including auxin, ABA, ethylene, zeatin and jasmonic acid (Table 3). These genes may underlie the plant architecture, leaf shape, and disease resistance traits of ZC108 as well.

ESTs probe sets corresponded to abiotic stress-related transcription factors, encoding proteins in the AP2-EREBP, NAC, MYB, ZF-HD, C2H2, MDAS, REM and bZIP families. MADS-box transcription factor expression in response to the infection was different between ZC108 and LJ43 (Fig 4). After infection, a *MADS* gene defined by homology to AtAGL14 (AT4G11880) was down-regulated in LJ43 but up-regulated in ZC108, suggesting it might play an important role in regulating tea plant response to *Colletotrichum camelliae*. *MADS* is therefore a promising candidate gene for future studies of the molecular mechanisms underlying AD response.

Genes involved in phenylpropanoid biosynthesis were differentially expressed between ZC108 and LJ43 (Fig 3B). These differences may confer disease resistance to ZC108. Saravanakumar and colleagues have shown that accumulation of peroxidase and PAL increases in response to pathogen infection in the tea plant [4]. PAL is one of the most extensively studied enzymes in the plant biotic and abiotic stress response pathways [39]. Consistent with these previous findings, the present study demonstrated that PAL transcription increased after infection. All three *PAL* probes in our microarray were significantly up-regulated in ZC108 (by 2.29, 4.52, and 5.67-fold). Furthermore, *PAL* was up-regulated in ZC108 regardless of whether it was inoculated with *Colletotrichum camelliae* (Fig 4). These results suggest that PAL might be the key gene responsible for higher resistance to *Colletotrichum camelliae* in ZC108.

Notably, after inoculation, POD, ALDH, beta-glucosidase, and two CAD genes showed higher expression in ZC108 than LJ43 (Fig 4). CAD is a key enzyme in lignin biosynthesis. Deng *et al*. showed that one CAD, *CsCAD3*, was strongly up-regulated after *E*. *oblique* attack and mechanical damage, and three *CsCADs* responded to treatment with the defense-related hormones methyljasmonate (MeJA) and salicylic acid (SA) [40]. In the present study, the expression of two *CADs* was higher in ZC108 than LJ43 after *Colletotrichum camelliae* inoculation, suggesting that the CAD is important for ZC108 resistance (Fig 4). Our study indicates that beta-glucosidase may be involved in AD resistance, consistent with previous reports that it is important in the plant defense response [43]. In addition, VpALDH2B4 may be beneficial to the plant under conditions of abiotic stress. In our study, the expression of ALDHs were significantly up-regulated in both normal and *Colletotrichum camelliae*-infected ZC108 (Table 2 and Fig 4). This result agrees with previous reports and reveals that CsALDHs may be important for AD resistance. POD plays a key role in plant defense through lignin formation, cross-linking of cell wall components, phytoalexin synthesis, and metabolism of reactive oxygen species (ROS) reactive nitrogen species (RNS), and auxin [59]. In *Arabidopsis*, the French bean peroxidase type 1 (FBP1) knockdown line exhibits an impaired oxidative burst and is more susceptible than wild type to both fungal and bacterial pathogens [41]. Furthermore, the AtPOD33 knockdown line is more susceptible to *Pseudomonas syringae* than wild type plants [42]. In this study, several PODs were differentially expressed in ZC108. Most were down-regulated in the absence of pathogen. However, after *Colletotrichum camelliae* infection, *POD* was up-regulated in ZC108 compared to LJ43 (Fig 4). These results demonstrate that POD can be regulated in response to *Colletotrichum camelliae* infection in the tea plant. Beta-glucosidase is another important component of the defense response, and our study also indicated it may be involved in AD resistance [43]. Phenylpropanoid metabolism gives rise to numerous compounds, including lignin. Down-regulating lignin pathway genes can alter metabolic flux and differentially affect the biosynthesis of other secondary compounds involved in plant-pathogen interaction [18]. In this study, CCR was down-regulated and CADs were up-regulated in ZC108, indicating that the biosynthesis of lignin and secondary compounds may be altered in this cultivar. Together, these results demonstrate that the phenylpropanoid pathway plays a vital role in AD resistance in ZC108. Altered expression levels of genes involved in the phenylpropanoid pathway may underlie ZC108 resistance to *Colletotrichum camelliae*.

Several studies have reported that genes involved in flavonoid biosynthesis (CHS, DFR and CHI) are also involved in the response to AD [8, 26]. However, CHS, DFR and CHI transcription did not significantly change between ZC108 and LJ43, regardless of *Colletotrichum camelliae* inoculation (data not shown).

Levels of secondary metabolites, such as phenylpropanoid compounds, are controlled in response to environmental cues and play an important role in host defense [10, 60]. We have measured tea polyphenol (TP) content in leaves of ZC108 and LJ43, and found that the leaf TP content was increased in both ZC108 and LJ43 after inoculation, indicating that TP played an important role in resistance to AD in tea plant (S5 Fig). Compared to LJ43, the TP content in ZC108 was higher than LJ43 at the 24 h post inoculation, however, it was not significant (S5 Fig). Flavonoids, isoflavonoids, anthocyanins, phytoalexins and lignins belong to phenolic compounds. Although there was no significant difference between TP content in ZC108 and LJ43 after inoculation, it will be of great interest to examine other phenolic compounds, e.g. flavonoid, phytoalexins and lignin content as a possible mechanism for ZC108 pathogen resistance in future studies. Understanding defense mechanisms in anthracnose-resistant tea plants is important for plant breeding. Although differentially expressed genes in the transcriptomes of LJ43 and ZC108 were identified, we did not compare these transcriptomes after inoculation. This will be important to examine in the future to further elucidate the molecular mechanisms underlying AD resistance. ZC108 was selected from LJ43 cuttings that underwent Co^60^γ-ray radiation. Genes in ZC108 which were mutated to substantially change plant architecture and biochemical composition are still unknown. In future studies, identification of the mutated genes will contribute to our understanding of the molecular mechanisms underlying plant architecture and facilitate breeding elite varieties.

Tea plant breeding is very difficult due to very long procudure, very low breeding efficiency and natural mutant rate. So find a higher effective breeding method is very important for tea plant improvement. Radiaction mutation breeding is an effective breeding method in plant, especially some perennial species, such as tea plant. Radiaction is not only make some functional gene mutation, but also make epigenetic variations at DNA level [61, 62]. So in the following studies, we’ll conduct some projects on DNA epigenetic effects and functional genomics to illustrate the mechanisms of the phenotypic variation between ZC108 and LJ43 based on the whole genome sequnces of tea plant be sequenced. And this will be very useful for establishment a effective radiation mutant breeding method in tea plant.

In summary, we identified ZC108 as an anthracnose-resistant tea plant cultivar. We compared plant morphology, biochemistry, and transcriptomes of ZC108 and LJ43 and identified many differences. The levels of genes involved in plant hormone biosynthesis and signaling, plant-pathogen interaction, and secondary metabolic pathways (including phenylpropanoid biosynthesis), were changed in ZC108. We selected genes to examine in infected and uninfected tea plant leaves and confirmed that several genes may contribute to anthracnose disease tolerance in ZC108. These include MADS-box transcription factor and genes involved in phenylpropanoid metabolism (CAD, CCR, POD, beta-glucosidase, ALDH and PAL). Additional studies to measure phenylpropanoid compound content and transcriptomes after inoculation will expand our understanding of the mechanisms of AD resistance.

## Supporting Information

S1 Fig Expression analysis of 7 transcription factors in response to *Colletotrichum camelliae* infection. Gene expression analysis were examined by qRT-PCR using cDNA from leaves of three-year-old LJ43 and ZC108 after inoculated by *Colletotrichum camelliae* for 0, 24 and 72 h. All of the qRT-PCR values were expressed relative to the expression level of LJ43-0h (control-set to 1.0). The data of ZC108 from microarray was expressed relative to the transcript abundance of LJ43 from microarray (control-set to 1.0). Data shown as the mean ±SD (*n* = 3).
(TIF)

S2 Fig Expression analysis of 6 plant-pathogen interaction-related genes in response to *Colletotrichum camelliae* infection. (TIF)

S3 Fig Expression analysis of 7 plant hormone-related genes in response to *Colletotrichum camelliae* infection. (TIF)

S4 Fig Expression analysis of 7 genes involved in phenylpropanoid biosynthesis in response to *Colletotrichum camelliae* infection. (TIF)

S5 Fig Tea polyphenols content in the leaves of LJ43 and ZC108 at 0 and 24 h post infection. Three-year-old plants were grown in the green house for 1 month before *in vivo* inoculation. The first to third mature leaves from the top of LJ43 and ZC108 without inoculation and 24 h post inoculation were sampled. Data shown as the mean ±SD (*n* = 3).
(TIF)

S1 Table Primer sequences for quantitative RT-PCR. (DOCX)

S2 Table Genes differentially expressed between ZC108 and LJ43 mapped to 236 pathways. (XLS)

## Abbreviations

AD: anthracnose disease
ALDH: coniferyl-aldehyde dehydrogenase
ARF: auxin response factor
AUX/IAA: auxin-responsive protein IAA
CAD: cinnamyl-alcohol dehydrogenase
CCR: cinnamoyl-CoA reductase
CHI: chalcone isomerase
CHS: chalcone synthase
CKX: cytokinin dehydrogenase
DFR: dihydroflavonol 4-reductase
EC: enzyme commission
GO: gene ontology
HSP: heat shock protein
KEGG: kyoto encyclopedia of genes and genomes
FC: fold change
FDR: false discovery rate
NBS-LRR: nucleotide-binding site (NBS) and leucine-reach repeats (LRR)
PAL: phenylalanine ammonia-lyase
PTB: polypyrimidine tract-binding protein
POD: peroxidase
TP: tea polyphenol
