# Supplementary material for: Transcriptome Analysis of an Anthracnose-Resistant Tea Plant Cultivar Reveals Genes Associated with Resistance to Colletotrichum camelliae
Source: PLoS One. 2016 Feb 5;11(2):e0148535. doi: 10.1371/journal.pone.0148535 (PMC4743920; doi:10.1371/journal.pone.0148535)
Supplement: S1 Table — (DOCX) [file pone.0148535.s006.docx]

**S1 Table Primer sequences for quantitative RT-PCR.**

| **Genes** | **Forward Primer (5’ - 3’)** | **Reverse Primer (5’- 3’)** |
| --- | --- | --- |
| **CsPTB** | TGACCAAGCACACTCCACACTATCG | TGCCCCCTTATCATCATCCACAA |
| **CUST_33289** | CGGAAGTGGCGGAAAAGAGG | TTCAATCTTCTGTGACATGG |
| **CUST_506** | CACCCTGTTAACTGGTGGCA | CTCGTGATTGCCTCGTCGAT |
| **CUST_13516** | GCACTGGATCACCTTGAACG | AACTGTAGCCATAGAAGAGC |
| **CUST_19468** | AGCTTTGGGTGGTGTCATGT | TAGCTCAACTCCCAGCTCAAG |
| **CUST_10940** | GGAATGGCAGATTCCACAGT | GTGACCCATTCGGAGTTCTG |
| **CUST_3374** | ACAGGAGAGAGAATTAGATCAC | GTGCACCATTCCAATCCTTGAG |
| **CUST_15181** | GTGCAACAAATGCCCCCAGA | TTCTGTAGGAAGAGCGTGGAG |
| **CUST_18562** | TGGTTTGGGGAAGACCACTC | CTGCCAACATCTTCCTTCCTCT |
| **CUST_1834** | CAAAGCAGGCAGCCTAAACC | CCCTCATCTTCCTCACTTTC |
| **CUST_4560** | CCACCTCATCATCAGATGGT | CCTTGAGGACTCTTCTCTTG |
| **CUST_12** | TGAGACTCCGGAAAGTCTCT | TTTGCCAGACTCTCCCATTC |
| **CUST_8204** | AGATCACAGCACCAGACATC | TCGGCGACATAGACAGCGTG |
| **CUST_53112** | ACTCCAAATTGACCCCTACC | AGAGACCATACTGGGTAAGC |
| **CUST_767** | GAGCATGGAGTTGAATGATG | CTGGTCTTCAAAGATGTGGC |
| **CUST_16624** | TCCGCTATCGTACTTGAACC | AATGGTGAGACCACCCATTG |
| **CUST_3691** | GATGGCTGCTTGGTCGTATG | GTGGGGTTCCTGGGTATCTG |
| **CUST_4471** | CAGCCGACTATGCTTCAGCT | GACCAGAAGTCACTTCGTCG |
| **CUST_12149** | GCTAGTGATCGAGTCATTTG | CGGCCAAAGATTTGAGGTTG |
| **CUST_2448** | AGGAGCCCCATCCATACACA | GGTTGGCCACCAGTTAGGTC |
| **CUST_42643** | CGATCCTCATCGATGGTCCT | AGCTGTGTCTTGGCATCATC |
| **CUST_36442** | GCAGCAGAACACAACATTTC | CCAATGTCAATTACAAATCG |
| **CUST_19864** | AGATTGAGCCATGGGTGTGG | TACCTGCTTGCTTGCTTGGT |
| **CUST_2483** | ACCAAGTGCAAGGATGAAAC | ACCGGTGTGAACTCCAAACC |
| **CUST_50500** | CGTGTGCAATTCGTTGCAGT | GCCCGCTGGAATTTTCTACAC |
| **CUST_8238** | GAGCCATGACTTGCTTTCAG | TCACCCGGTTCTTGGAGAAG |
| **CUST_18102** | ACTATGTTATAGTCATCAGC | ATGGCCAAGTGGTTCATTCC |
| **CUST_26291** | CGAGACGTTTGCTTTCGAGG | TTGCTAATGAGTTCACGGAG |
